# Supplementary material for: Dual targeting of RET and SRC synergizes in RET fusion‐positive cancer cells
Source: Mol Oncol. 2025 Nov 5;20(4):1041–60. doi: 10.1002/1878-0261.70155 (PMC13060644; doi:10.1002/1878-0261.70155)
Supplement: Supplementary file 1 — Fig. S1. Combination effects of RET tyrosine kinase inhibitors (TKIs) with dasatinib in RET + cancer cells. Fig. S2. SRC‐dependency of synergistic effects between RET tyrosine kinase inhibitors (TKIs) and dasatinib in LC‐2/Ad RET + non‐small cell lung cancer (NSCLC) cells. Fig. S3. Protein–protein network analysis of proteomics data in RET + CUTO32 non‐small cell lung cancer (NSCLC) cells. Fig. S4. Effects of selective inhibitions of AKT, mTOR and PAK. Fig. S5. Silencing of PAK1 in RET + LC2/Ad non‐small cell lung cancer (NSCLC) cells. Fig. S6. Combination effects of RET tyrosine kinase inhibitors (TKIs) with eCF506 in RET + cancer cells. [file MOL2-20-1041-s003.pdf]

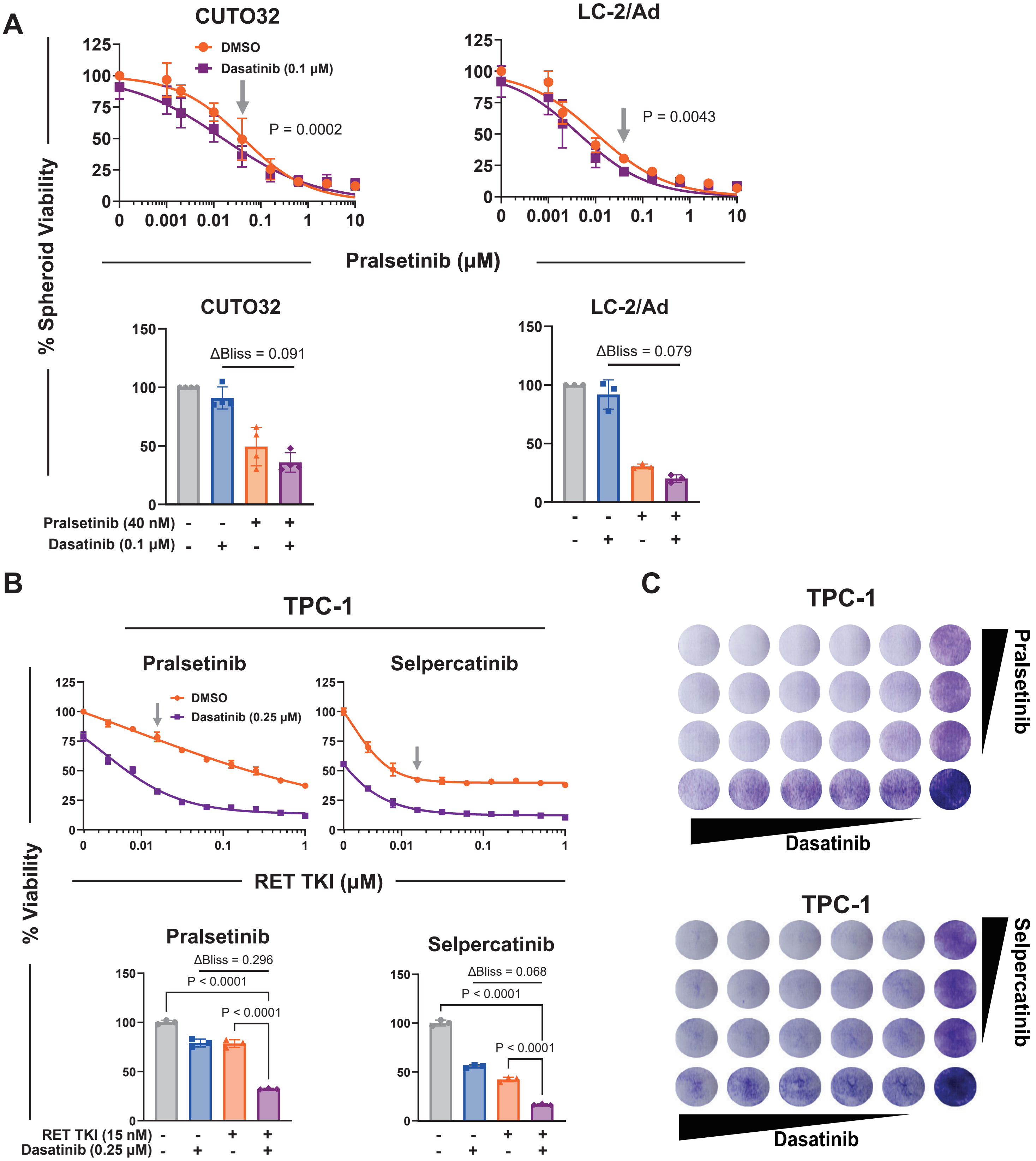

Supplementary Figure S1. Combination effects of RET tyrosine kinase inhibitors (TKIs) with dasatinib in *RET*⁺ cancer cells. (A) 3D cell viability in CUTO32 (n = 4) and LC-2/Ad (n = 3) spheroids after treatment with pralsetinib, dasatinib (0.1 µM) or the combination thereof. IC50 values were calculated by nonlinear regression analysis of dose response curves, and the extra sum-of-squares F test was used to determine statistical significance of IC50 differences between single agent pralsetinib and combination treatment groups (top). Cell viability-based Bliss synergy analysis of CUTO32 and LC-2/Ad cells in 3D culture at select concentration of pralsetinib (40 nM, see grey arrows above) (bottom). (B) Cell viability of *RET*⁺ TPC-1 papillary thyroid cancer (PTC) cells (n = 3) upon treatment with RET TKIs (pralsetinib, selpercatinib) and dasatinib (0.25 µM) for 3 days (Top). Cell viability and Bliss synergy analysis of TPC-1 cells at single concentration of RET TKIs (15 nM) (Bottom). (C) Clonogenic survival of TPC-1 cells after treatment with RET TKIs and dasatinib for 7 days (n = 3). Cells were treated with 2-fold serial dilutions of each drug starting from 2.5 µM. Statistical significance was determined by one-way ANOVA with Tukey's multiple comparisons. Results are shown as mean ± SD.

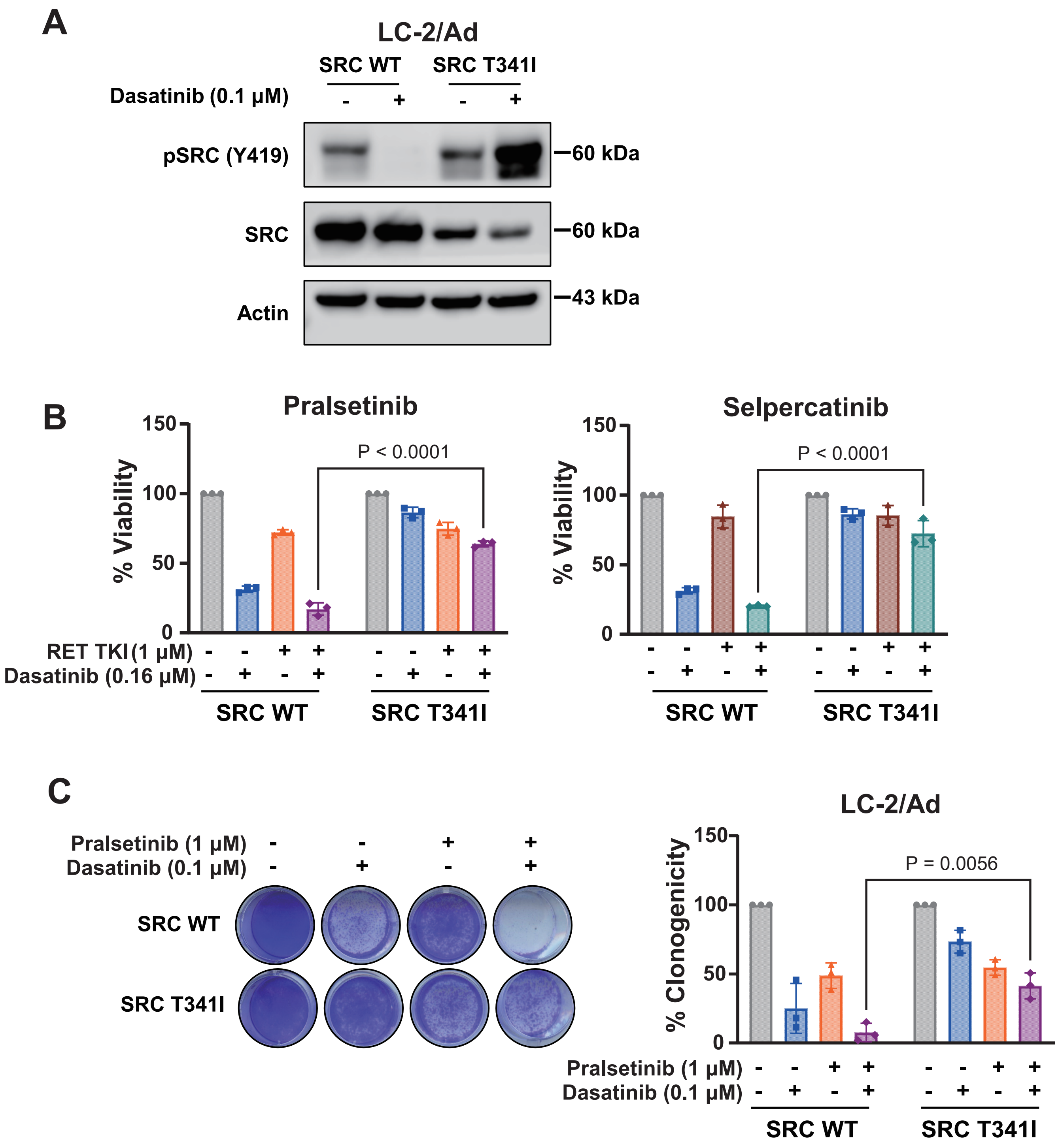

Supplementary Figure S2. SRC-dependency of synergistic effects between RET tyrosine kinase inhibitors (TKIs) and dasatinib in LC-2/Ad *RET*⁺ non-small cell lung cancer (NSCLC) cells. (A) SRC autophosphorylation in LC-2/Ad cells expressing SRC wild-type (WT) or SRC T341I gatekeeper mutation upon treatment with dasatinib (0.1 μM) for 3 hours ($n = 3$). (B) Cell viability of LC-2/Ad cells expressing SRC WT or SRC T341I gate-keeper mutation after combined treatment with dasatinib (0.16 μM) and RET TKIs pralse-tinib and selpercatinib (both 1 μM) for 3 days ($n = 3$). (C) Clonogenic survival and quantification of LC-2/Ad expressing SRC WT or SRC T341I gatekeeper mutation upon treatment with pralsetinib (1 μM), dasatinib (0.1 μM) or combination thereof for 7 days ($n = 3$). Statistical significance was determined by one-way ANOVA with Tukey's multiple comparisons. Results are shown as mean ± SD.

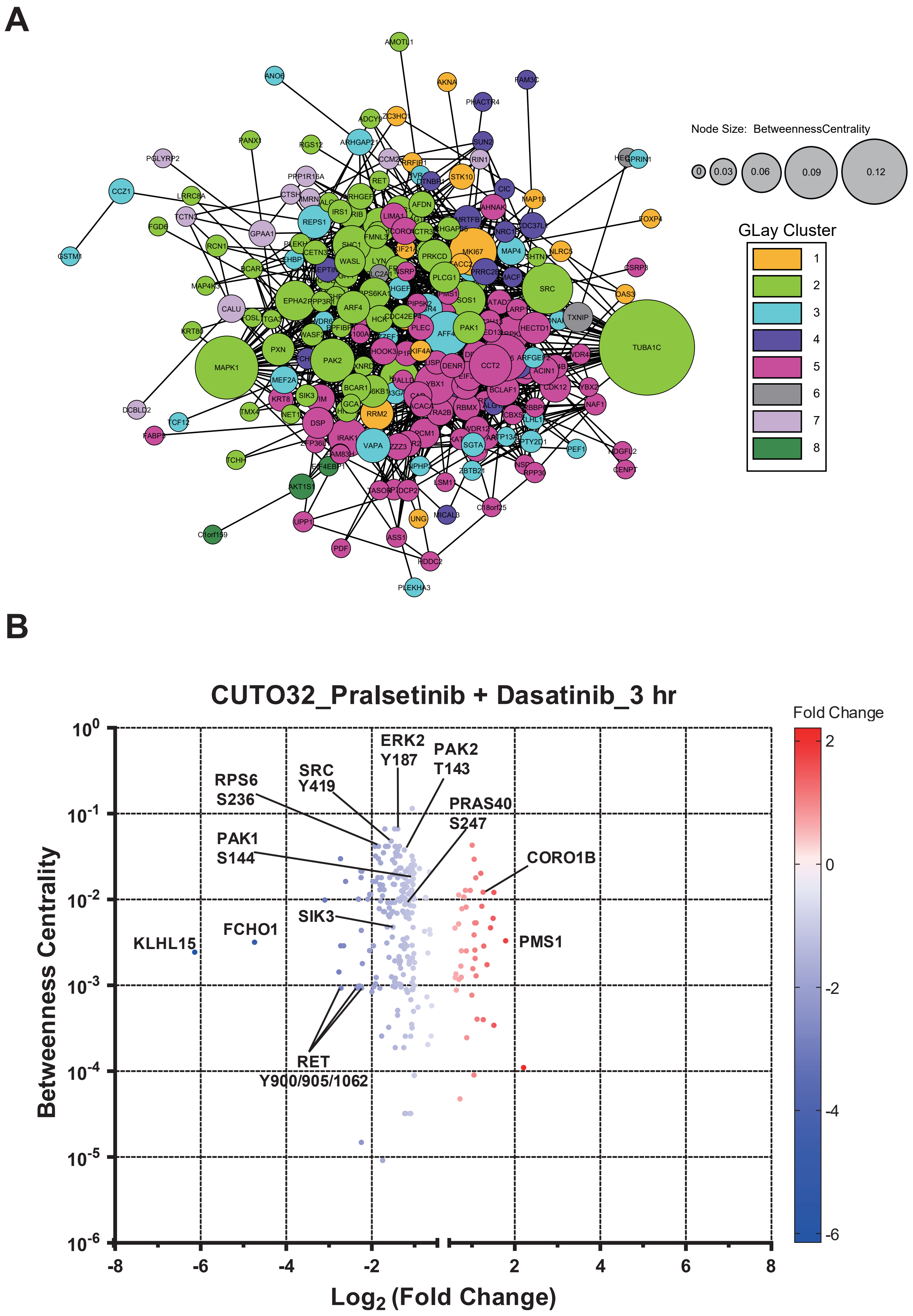

Supplementary Figure S3. Protein-protein network analysis of proteomics data in *RET*+ CUTO32 non-small cell lung cancer (NSCLC) cells. (A) STRING-based protein-protein network analysis and GLay-based subclustering of RET+ CUTO32 NSCLC cells after co-treatment with pralsetinib (1 μM) and dasatinib (0.1 μM) for 3 hours. All regulated proteins from phosphoproteomics and expression proteomics data were combined. (B) Scatter plot based on fold changes (phosphorylation or total expression) and betweenness centrality of proteins significantly regulated by combination treatment. Color gradient represents log2 fold change of signals.

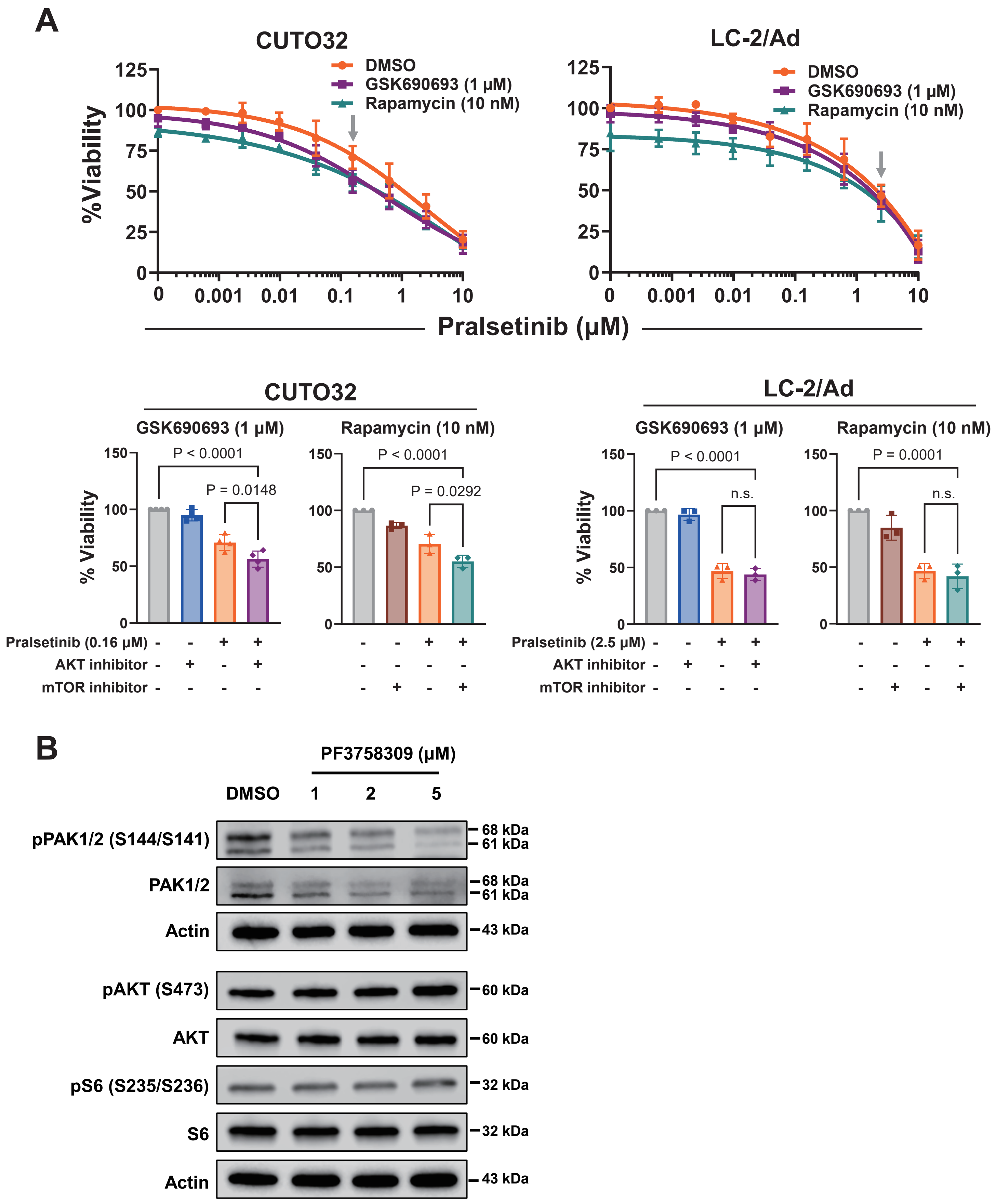

Supplementary Figure S4. Effects of selective inhibitions of AKT, mTOR and PAK. (A) (Top) Cell viability of *RET*+ non-small cell lung cancer (NSCLC) cells upon treatment with pralsetinib combined with the AKT inhibitor GSK690693 (1 μM, *n* = 4 for CUTO32 and *n* = 3 for LC-2/Ad) or the mTOR inhibitor rapamycin (10 nM, *n* = 3). (Bottom) Cell viability at the single concentration of pralsetinib (CUTO32: 0.16 μM; LC-2/Ad: 2.5 μM). (B) Phosphorylation of PAK1/2, AKT and S6 in CUTO32 cells after treatment with PF-3758309 at indicated concentrations for 3 hours (*n* = 2). Statistical significance was determined by one-way ANOVA with Tukey's multiple comparisons. Results are shown as mean ± SD.

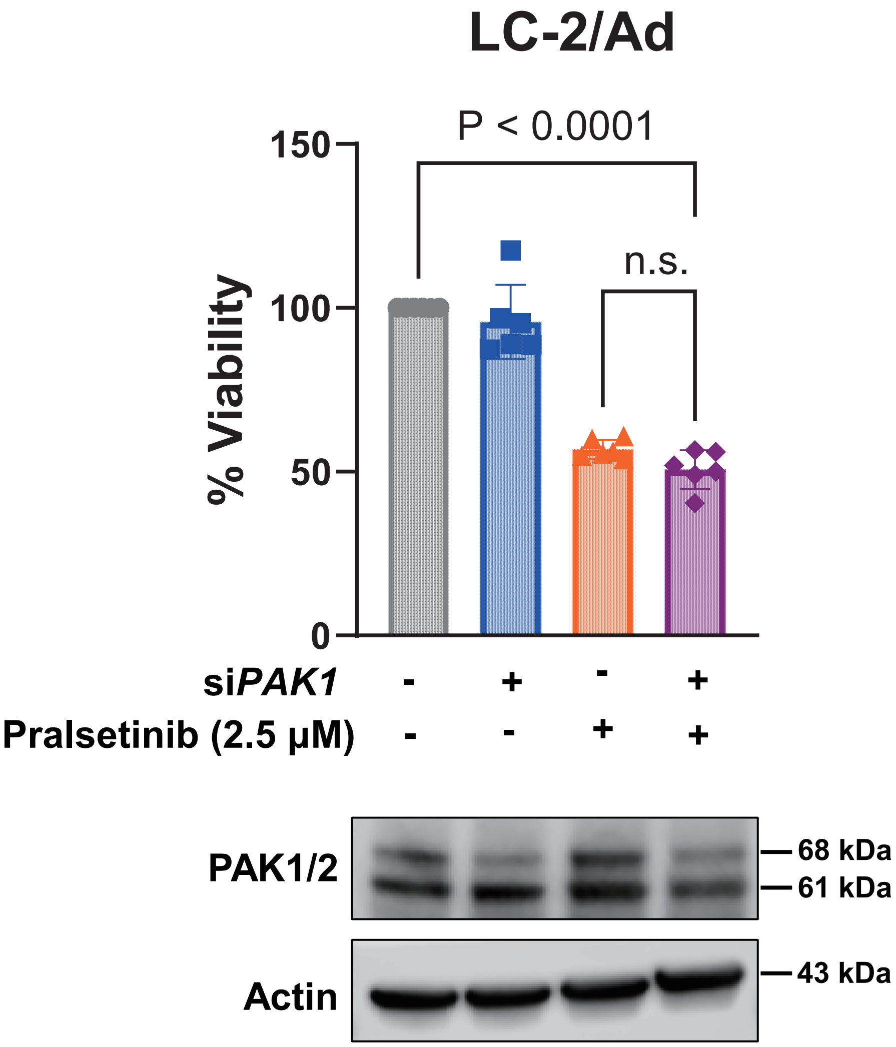

Supplementary Figure S5. Silencing of PAK1 in *RET*+ LC2/Ad non-small cell lung cancer (NSCLC) cells. (Top) Cell viability of LC-2/Ad cells (*n* = 6) after siRNA-mediated PAK1 knockdown (4 days) and pralsetinib treatment (2.5 μM, 3 days). (Bottom) Western blotting of PAK1 knockdown in LC-2/ad cells (*n* = 5). Statistical significance was determined by one-way ANOVA with Tukey's multiple comparisons; n.s., not significant. Results are shown as mean ± SD.

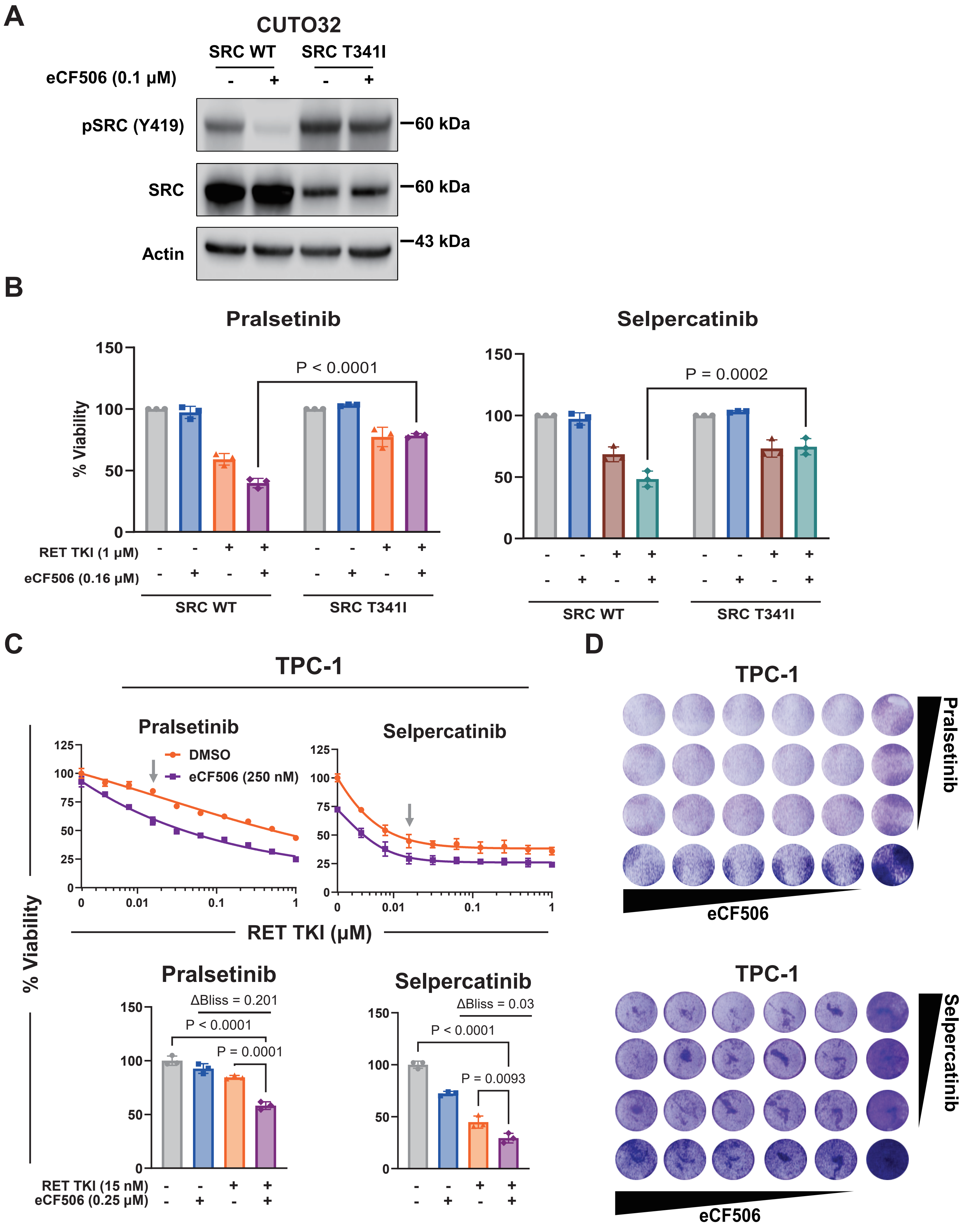

Supplementary Figure S6. Combination effects of RET tyrosine kinase inhibitors (TKIs) with eCF506 in *RET*+ cancer cells. (A) SRC autophosphorylation in CUTO32 cells expressing SRC WT or SRC T341I gatekeeper mutation upon treatment with eCF506 (0.1 µM) for 3 hours (*n* = 3). (B) Cell viability of CUTO32 cells expressing SRC WT or SRC T341I gatekeeper mutation after combined treatment with eCF506 (0.16 µM) and RET TKIs pralsetinib and selpercatinib (both 1 µM) for 3 days (*n* = 3). (C) (Top) Cell viability of TPC-1 cells upon treatment with RET TKIs alone and combined with eCF506 (0.25 µM) for 3 days (*n* = 3). (Bottom) Cell viability and Bliss synergy analysis of TPC-1 cells at single concentration of RET TKIs (15 nM). (D) Clonogenic survival of TPC-1 cells after treatment with RET TKIs, eCF506 or their combination for 7 days (*n* = 3). Cells were treated with 2-fold serial dilutions of each drug starting from 2.5 µM. Statistical significance was determined by one-way ANOVA with Tukey's multiple comparisons. Results are shown as mean ± SD.
